# Minimal brain PBPK model to support the preclinical and clinical development of antibody therapeutics for CNS diseases

**DOI:** 10.1007/s10928-021-09776-7

**Published:** 2021-08-10

**Authors:** Peter Bloomingdale, Suruchi Bakshi, Christian Maass, Eline van Maanen, Cesar Pichardo-Almarza, Daniela Bumbaca Yadav, Piet van der Graaf, Nitin Mehrotra

**Affiliations:** 1 Pharmacokinetics, Pharmacodynamics, and Drug Metabolism, Merck & Co. Inc., Boston, MA USA; 2 Certara QSP, Oss, The Netherlands; 3 Present Address: esqLABS GmbH, Berlin, Germany; 4 Certara IDD, Oss, The Netherlands; 5 Certara QSP, Canterbury, UK

**Keywords:** PBPK, Antibody, Neuroscience, Pharmacokinetics, Drug development

## Abstract

There are several antibody therapeutics in preclinical and clinical development, industry-wide, for the treatment of central nervous system (CNS) disorders. Due to the limited permeability of antibodies across brain barriers, the quantitative understanding of antibody exposure in the CNS is important for the design of antibody drug characteristics and determining appropriate dosing regimens. We have developed a minimal physiologically-based pharmacokinetic (mPBPK) model of the brain for antibody therapeutics, which was reduced from an existing multi-species platform brain PBPK model. All non-brain compartments were combined into a single tissue compartment and cerebral spinal fluid (CSF) compartments were combined into a single CSF compartment. The mPBPK model contains 16 differential equations, compared to 100 in the original PBPK model, and improved simulation speed approximately 11-fold. Area under the curve ratios for minimal versus full PBPK models were close to 1 across species for both brain and plasma compartments, which indicates the reduced model simulations are similar to those of the original model. The minimal model retained detailed physiological processes of the brain while not significantly affecting model predictability, which supports the law of parsimony in the context of balancing model complexity with added predictive power. The minimal model has a variety of applications for supporting the preclinical development of antibody therapeutics and can be expanded to include target information for evaluating target engagement to inform clinical dose selection.

**Supplementary Information:**

The online version contains supplementary material available at 10.1007/s10928-021-09776-7.

## Introduction

Over the past few decades, there has been a surge of antibody therapeutics that have made their way into clinical practice [1]. The first FDA approved antibody therapeutic, approved in 1986, was muromonab, which is an anti-CD3 antibody used for organ transplantation to prevent graft-versus-host disease. As of December 2019, there were at least 570 antibody therapeutics clinically investigated and 79 that have been approved by the FDA [2]. The majority of therapeutic antibodies have been developed for the treatment of cancer and immune-related diseases. A few antibodies have been FDA approved for the treatment of neurological and CNS disorders, such as multiple sclerosis, migraine, and neuromyelitis optica [3–5]. However, the site of action for most of these therapeutic antibodies is peripheral and they do not need to cross brain barrier for pharmacological effects.

There has been increased interest and significant investments made to develop therapeutic antibodies as a passive immunotherapy strategy for the treatment of neurodegenerative diseases [6]. However, to date, there has not been any clinical success and no antibody therapeutic has been fully approved for the treatment of a neurodegenerative disease. On June 7th of 2021, the FDA granted accelerated approval of aducanumab, a beta-amyloid (Aβ) antibody for the treatment of Alzheimer's disease. Disrupted proteostasis is a hallmark of neurodegeneration as there exists an underlying protein aggregation and clearance problem associated with neurodegenerative disease. Alzheimer’s disease (AD) is the most prevalent neurodegenerative diseases affecting over 46 million people worldwide [7]. There are several antibody therapeutics under clinical development targeting Aβ and tau for the treatment of AD [8]. The hypothesis behind this proposed treatment strategy is that antibodies will bind to extracellular forms of the pathological proteins, which could facilitate clearance and prevent protein aggregation, neuron-to-neuron transmission, and neuronal damage. Numerous phase 2/3 clinical trials investigating anti-Aβ antibodies for the treatment of AD were negative, as clinical endpoints of cognition (e.g. ADAS-Cog and CDR-SB) were not improved [9]. Tau-targeting antibodies are in early clinical development (phase 1/2). In addition to AD, there are several other neurodegenerative diseases where therapeutic antibodies are being investigated, such as Parkinson’s disease, tauopathies, amyotrophic lateral sclerosis, and Huntington’s disease.

One of the main challenges facing the potential utility of biologics for the treatment of CNS diseases is achieving brain exposures that is above a therapeutic threshold [10]. The physicochemical properties of biologics, primarily their molecular size, limits their ability to cross brain barriers, which results in low CNS exposure. Physical barriers, such as the blood–brain-barrier (BBB) and blood–cerebral-spinal-fluid-barrier (BCSFB), are designed by nature to be highly regulated gateways in the body in order to protect the brain from toxins and pathogens. The BBB is the barrier interfacing systemic circulation and brain parenchyma. The components that make up the BBB, known as the neurovascular unit, are vascular endothelial cells forming tight junctions, basal lamina, pericytes, astrocytes, microglia, and neurons [11]. The BCSFB is the barrier that interfaces systemic circulation with ventricular CSF. The components that make up the BCSFB are vascular endothelial cells, basement membrane, and epithelial cells forming tight junctions [12]. Once in the ventricular system, antibodies need to cross the ventricular barrier, consisting of an ependymal cell layer, to reach the brain parenchyma. The relative leakiness of the BCSFB compared to the BBB is an attractive hypothesis how antibodies enter the brain. However, the routes of antibody disposition into the brain and their relative contributions remains unclear.

Ability to predict the level of antibody exposure in the human brain required for adequate target engagement is critical to clinical drug development programs. This was recently exemplified when aducanumab was discontinued following a phase III futility analysis, but subsequent data from patients exposed to a higher dose for a longer period of time suggested potential efficacy [13]. The failure to demonstrate the efficacy of aducanumab from prior trials has sparked interest in the AD community for conducting another clinical trial using a high dose [14]. 

Pharmacokinetic modeling is used for understanding the time course of drug exposure through mathematically describing kinetic processes that govern drug absorption, distribution, and elimination. Over the past couple of decades there has been a shift from classical compartmental pharmacokinetic models towards more mechanistic models that include detailed physiological processes, compartments that represent specific organs, and realistic parameter values that are constrained by known physiology. These types of models are known as physiologically-based pharmacokinetic (PBPK) models. PBPK models date back to the 1930s, but have become popularized due to the applications in drug discovery and development [15]. There has been an increased interest in reducing PBPK models to less complex structures, known as minimal PBPK (mPBPK) models, in order to increases transparency and enhance the ease of application, whilst retaining the key mechanistic features and behaviors [16].

As far as we are aware, a mPBPK approach has not been reported yet for antibodies targeting the CNS. Therefore, we have developed a mPBPK model of the brain for antibody therapeutics, based on an existing multi-species platform brain PBPK model [17].

## Methods

### Structural model reduction and reparameterization

A mPBPK model of the brain was constructed through the stepwise reduction of a previously developed PBPK model, further referred to as the “*full*” model [17]. The reduction approach was implemented to reduce model complexity, while conserving physiological details and model predictability. Fourteen compartments representing all non-brain organs were combined into a single tissue compartment, which is divided into three subcompartments (vascular, endosomal, and interstitium). The four CSF compartments (lateral ventricle, third-fourth ventricle, cisterna magna, and subarachnoid space) were combined into a single CSF compartment. Brain vascular, endosomal, and interstitial spaces remained the same. Model parameters are provided in Table 1.

**Table 1:** Minimal brain PBPK model parameters

Parameter	Units	Mouse	Rat	Monkey	Human
FcRn	M	4.98E−05	4.98E−05	4.98E−05	4.98E−05
k_deg_	1/h	26.6	26.6	26.6	26.6
FR	–	0.715	0.715	0.715	0.715
FR_B_	–	0.715	0.715	0.715	0.715
kon_FcRn_	1/M/h	8.06E+07	8.00E+08	7.92E+08	5.59E+08
koff_FcRn_	1/h	6.55	144	46.8	23.9
V_P_	L	9.44E−04	0.00906	0.187	3.13
V_Tv_	L	8.06E−04	0.00789	0.151	1.68
V_Te_	L	1.28E−04	0.00132	0.0286	0.335
V_Ti_	L	0.00482	0.0483	0.976	11.1
V_Bv_	L	1.07E−05	5.02E−05	0.00207	0.0319
V_BE_BBB_	L	2.09E−06	9.82E−06	4.27E−04	0.00659
V_BE_BCSFB_	L	3.37E−07	1.58E−06	4.27E−05	6.59E−04
V_Bi_	L	8.73E−05	4.10E−04	0.0169	0.261
V_CSF_	L	1.93E−05	2.97E−04	0.00926	0.143
V_L_	L	1.13E−04	0.00115	0.0251	0.274
Q_T_	L/h	0.361	2.88	20.9	160.5
Q_B_	L/h	0.0118	0.0653	1.51	21.5
L_T_	L/h	7.23E−04	0.00577	0.0419	0.321
L_B_	L/h	2.16E−05	1.62E−04	0.00369	0.0345
Q_B_ECF_	L/h	1.80E−06	3.00E−05	0.00123	0.0105
Q_B_CSF_	L/h	1.98E−05	1.32E−04	0.00246	0.0240
k_CLUPT_	1/h	0.550	0.550	0.550	0.550
k_CLUPB_	1/h	0.0195	0.0195	0.0195	0.0195
CLUP_T_	L/h	7.06E−05	7.26E−04	0.0157	0.184
CLUP_B_	L/h	4.74E−08	2.23E−07	9.19E−06	1.42E−04
CLUP_BBB_	L/h	4.08E−08	1.92E−07	8.35E−06	1.29E−04
CLUP_BCSFB_	L/h	6.58E−09	3.09E−08	8.35E−07	1.29E−05
σ_BBB_	–	1	1	1	1
σ_BCSFB_	–	0.9973	0.9973	0.9973	0.9973
σ_Tv_	–	0.9172	0.9212	0.9239	0.9233
σ_TL_	–	0.2	0.2	0.2	0.2
σ_ISF_	–	0.2	0.2	0.2	0.2
σ_CSF_	–	0.2	0.2	0.2	0.2
SA_BBB_	m^2^	0.0155	0.0155	17	17
SA_BCSFB_	m^2^	0.0025	0.0025	1.7	1.7
f_BBB_	–	0.861	0.861	0.909	0.909

All model parameter values were obtained from the original brain PBPK model described in Chang et al. [17]. Neonatal Fc receptor (FcRn), antibody endosomal degradation rate (k_deg_), fraction of antibody recycled (FR_B_), fraction of antibody recycled in brain (FR_B_), antibody-FcRn association rate (kon_FcRn_), antibody-FcRn complex dissociation rate (koff_FcRn_), plasma volume (V_P_), tissue vascular volume (V_Tv_), tissue endosomal volume (V_Te_), tissue interstitial volume (V_Ti_), brain vascular volume (V_Bv_), blood–brain-barrier endosomal volume (V_BE_BBB_), blood–CSF-barrier endosomal volume (V_BE_BCSFB_), brain interstitial volume (V_Bi_), brain CSF volume (V_CSF_), tissue blood flow (Q_T_), brain blood flow (Q_B_), tissue lymphatic flow (L_T_), brain lymphatic flow (L_B_), brain ECF flow (Q_B_ECF_), brain CSF flow (Q_B_CSF_), tissue uptake clearance rate (k_CLUPT_), brain uptake clearance rate (k_CLUPB_), tissue uptake clearance (CLUP_T_), brain uptake clearance (CLUP_B_), blood–brain barrier uptake clearance (CLUP_BBB_), blood–CSF-barrier uptake clearance (CLUP_BCSFB_), blood–brain-barrier reflection coefficient (σ_BBB_), blood–CSF-barrier reflection coefficient (σ_BCSFB_), tissue vascular reflection coefficient (σ_Tv_), tissue lymph reflection coefficient (σ_TL_), brain ISF reflection coefficient (σ_ISF_), brain CSF reflection coefficient (σ_CSF_), blood–brain-barrier surface area (SA_BBB_), blood–CSF-barrier surface area (SA_BCSFB_)

Due to the structural reduction, a reparameterization of certain volumes, flow rates, endosomal uptake rates, and tissue reflection coefficients was required. Parameter values of the minimal model were either conserved or combined from the full model. Three parameters for tissue volumes, tissue vascular volume ${(V}_{{T}_{V}})$, tissue endosomal volume ${(V}_{{T}_{E}}),$ and tissue interstitial volume ${(V}_{{T}_{I}}),$ were calculated based upon the sum of the respective individual tissue compartments of the full model. For example, the calculation for ${V}_{{T}_{V}}$ is:

1 $${V}_{{T}_{V}}={V}_{{Lung}_{V}}+{V}_{{Heart}_{V}}+{V}_{{Kidney}_{V}}+{V}_{{Muscle}_{V}}+{V}_{{Skin}_{V}}+{V}_{{Adipose}_{V }}+{V}_{{Thymus}_{V}}+{V}_{S{Intestine}_{V}}+{V}_{L{Intestine}_{V}}+{V}_{{Spleen}_{V}}+{V}_{{Pancreas}_{V}}+{V}_{{Liver}_{V}}+{V}_{{Bone}_{V}}+{V}_{{Other}_{V}}$$

The same calculation was performed for ${V}_{{T}_{E}}$ and ${V}_{{T}_{I}}$. Brain vascular ${(V}_{{B}_{V}})$, endosomal $({V}_{{B}_{E}})$, and interstitial ${(V}_{{B}_{I}})$ volumes were unchanged. Four brain CSF volumes, lateral ventricle ${(V}_{LV})$, third-fourth ventricle ${(V}_{TFV})$, cisterna magna ${(V}_{CM})$, and subarachnoid space ${(V}_{SAS})$, were combined into a single CSF volume:

2 $${V}_{CSF}={V}_{LV}+{V}_{TFV}+{V}_{CM}+{V}_{SAS}$$

The plasma flow rate to tissue ${(Q}_{T})$ was calculated based on the difference between lung ${(Q}_{L})$ and brain ${(Q}_{B})$ plasma flow:

3 $${Q}_{T}={Q}_{L}-{Q}_{B}$$

Note that the total plasma flow is equivalent to the lung plasma flow. Tissue lymph flow $({L}_{T})$ was calculated as 0.2% of tissue plasma flow, equivalent to the original model:

4 $${L}_{T}={0.002\times Q}_{T}$$

The original model used three different values for tissue reflection coefficients based upon the leakiness of the organ vasculature, categorized as loose ${(\sigma }_{Loose}=0.85)$, medium ${(\sigma }_{Medium}=0.90)$, and tight ${(\sigma }_{Tight}=0.95)$. In order to conserve this feature, we obtained a single tissue reflection coefficient ${(\sigma }_{{T}_{V}})$ based upon the weighted average reflection coefficient for each organ:

5 $${\sigma }_{{T}_{V}}=\left({\sigma }_{Lung}\cdot {V}_{{Lung}_{V}}+{\sigma }_{Heart}\cdot {V}_{{Heart}_{V}}+{{\sigma }_{Kidney}\cdot V}_{{Kidney}_{V}}+{\sigma }_{Muscle}\cdot {V}_{{Muscle}_{V}}+{\sigma }_{Skin}\cdot {V}_{{Skin}_{V}}+{\sigma }_{Adipose}\cdot {V}_{{Adipose}_{V }}+{\sigma }_{Thymus}\cdot {V}_{{Thymus}_{V}}+{\sigma }_{SIntestine}\cdot {V}_{S{Intestine}_{V}}+{\sigma }_{LIntestine}\cdot {V}_{L{Intestine}_{V}}+{\sigma }_{Spleen}\cdot {V}_{{Spleen}_{V}}+{\sigma }_{Pancreas}\cdot {V}_{{Pancreas}_{V}}+{\sigma }_{Liver}\cdot {V}_{{Liver}_{V}}+{\sigma }_{Bone}\cdot {V}_{{Bone}_{V}}+{\sigma }_{Other}\cdot {V}_{{Other}_{V}}\right) / {V}_{{Tissue}_{V}}$$

A single value was used for the lymph reflection coefficients in all tissues in the full model, which was the same value used in the minimal model ${(\sigma }_{{T}_{L}}=0.2)$.

All clearances and rates were conserved between the full and minimal models. However, in an attempt to avoid confusing nomenclature going forward, we have redefined the symbol for uptake clearance rates from $${CL}_{UP}$$ to $${k}_{{CL}_{UP}}$$, since this value is a rate constant with units of inverse time and not units of flow, volume per time. Tissue uptake clearance ($${CL}_{{UP}_{T}})$$ was calculated as the product of tissue uptake clearance rate $$({k}_{{CL}_{{UP}_{T}}})$$ and total tissue endosomal volume $${(V}_{{T}_{E}}).$$

6 $${CL}_{{UP}_{T}}={k}_{{CL}_{{UP}_{T}}}\times {V}_{{T}_{E}}$$

The same calculation was used for brain uptake clearance ${(CL}_{{UP}_{B}})$, the product of brain uptake clearance rate $({k}_{{CL}_{{UP}_{B}}})$ and brain endosomal volume ${(V}_{{B}_{E}})$.

7 $${CL}_{{UP}_{B}}={k}_{{CL}_{{UP}_{B}}}\times {V}_{{B}_{E}}$$

Brain uptake clearance is split between antibody disposition across the BBB ${(CL}_{{UP}_{BBB}})$ and BCSFB ${(CL}_{{UP}_{BCSFB}})$, which is scaled based upon their relative surface areas (SA).

8 $${CL}_{{UP}_{B}}={CL}_{{UP}_{BBB}}+{CL}_{{UP}_{BCSFB}}$$

9 $${f}_{BBB}=\frac{{SA}_{BBB}}{{SA}_{BBB} +{ SA}_{BCSFB}}$$

10 $${CL}_{{UP}_{BBB}}={k}_{{CL}_{{UP}_{B}}}\times {V}_{{B}_{E}}\times {f}_{BBB}$$

11 $${CL}_{{UP}_{BCSFB}}={k}_{{CL}_{{UP}_{B}}}\times {V}_{{B}_{E}}\times \left(1-{f}_{BBB}\right)$$

where $${f}_{BBB}$$ is the fraction of drug disposition across the BBB, $${SA}_{BBB}$$ is the surface area of the BBB, and $${SA}_{BCSFB}$$ is the surface area of the BCSFB. The endosomal volume for the blood–brain-barrier ($${V}_{{B}_{{E}_{BBB}}})$$ is equal to the product of $${V}_{{B}_{E}}$$ and $${f}_{BBB}$$. The endosomal volume for the blood−CSF-barrier ($${V}_{{B}_{{E}_{BCSFB}}})$$ is equal to the product of $${V}_{{B}_{E}}$$ and $$\left(1-{f}_{BBB}\right)$$.

The proportionality constant called *LNLF*, originally described by Shah and Betts in 2012 [18], was used in the full model to calculate lymph flow. This parameter was removed from the model because it was found to be insensitive, as there were no changes in pharmacokinetic profiles upon changing this value. Total lymph flow was recalculated as the sum of tissue lymph flow $({L}_{T})$ and brain lymph flow $({L}_{B})$. Brain lymph flow was calculated as the sum of brain ISF and CSF flow:

12 $${L}_{B}={Q}_{{B}_{ECF}}+{Q}_{{B}_{CF}}$$

Hence, physiological lymph flow rates were used instead of an empirical proportionality constant.

### Comparison of minimal and full PBPK model simulations

Simulations were performed to assess the accuracy of the minimal model for capturing the behavior of the full model. Single ascending dose (1, 3, 10, 30, 100 mg/kg) simulations were performed for each species (mouse, rat, monkey, and human) up to 1000 h, using an output step size of 0.01 h. Doses were administered directly into the plasma compartment to emulate intravenous (IV) dosing. Predictions using the full and minimal models were overlaid for a direct comparison. Additionally, area under the concentration time curve (AUC) for plasma and CNS compartments were calculated for each simulated profile using the *trapz* function in MATLAB. The AUC ratio between the minimal and full model simulations were calculated:

13 $${AUC}_{Ratio}=\frac{{AUC}_{Minimal}}{{AUC}_{Full}}$$

An AUC ratio of 1 would indicate that predicted antibody exposure by the minimal model is identical to the full model. An AUC ratio of > 1 or < 1 indicates that predicted antibody exposure by the minimal model is greater than or less than the full model, respectively.

To determine the computational speed for executing the minimal and full PBPK models, the *tic*–*toc* function in MATLAB was implemented before (tic) and after (toc) calling the model function.

### Sensitivity analysis

A one-at-a-time sensitivity analysis method was utilized to assess the sensitivity of antibody exposure when modulating parameter values. Parameters were sampled from a log normal distribution (µ = 0, σ = 0.25), using the *lognrnd* function in MATLAB. 1000 and 100 parameter sets were generated for 27 and 25 parameters in the minimal and full model, respectively. Simulations were performed for each parameter set for a 70 kg human administered 10 mg/kg over 1000 h, using a step size of 1 h.

AUC was determined, using the *trapz* function in MATLAB, for three compartments: (1) plasma, (2) CSF, and (3) ISF. A metric of sensitivity (S), representing the percentage change in antibody exposure, was calculated as follows:

14 $${S}_{x} \left(\%\right)=\left(\frac{{AUC}_{Perturb}}{{AUC}_{Base}}-1\right)\times 100 \%$$

where S_x_ represents the percentage change in antibody exposure by modulation of parameter *x*. AUC_Base_ and AUC_Pertrub_ represent the area under the concentration–time curve before and after perturbation of parameter *x*, respectively. Figures were created in R, using *ggplot2*.

Total and tissue blood flow were excluded from the analysis for the full model, due to issues where simulations would take a long time and not complete. The lung was selected as the tissue to represent the sensitivity of the tissue vascular $({V}_{{T}_{V}})$, tissue endosomal volume $({V}_{{T}_{E}})$, and tissue interstitial volume $({V}_{{T}_{I}})$ for the full PBPK model.

## Results

### Brain minimal PBPK model

**Fig. 1:**
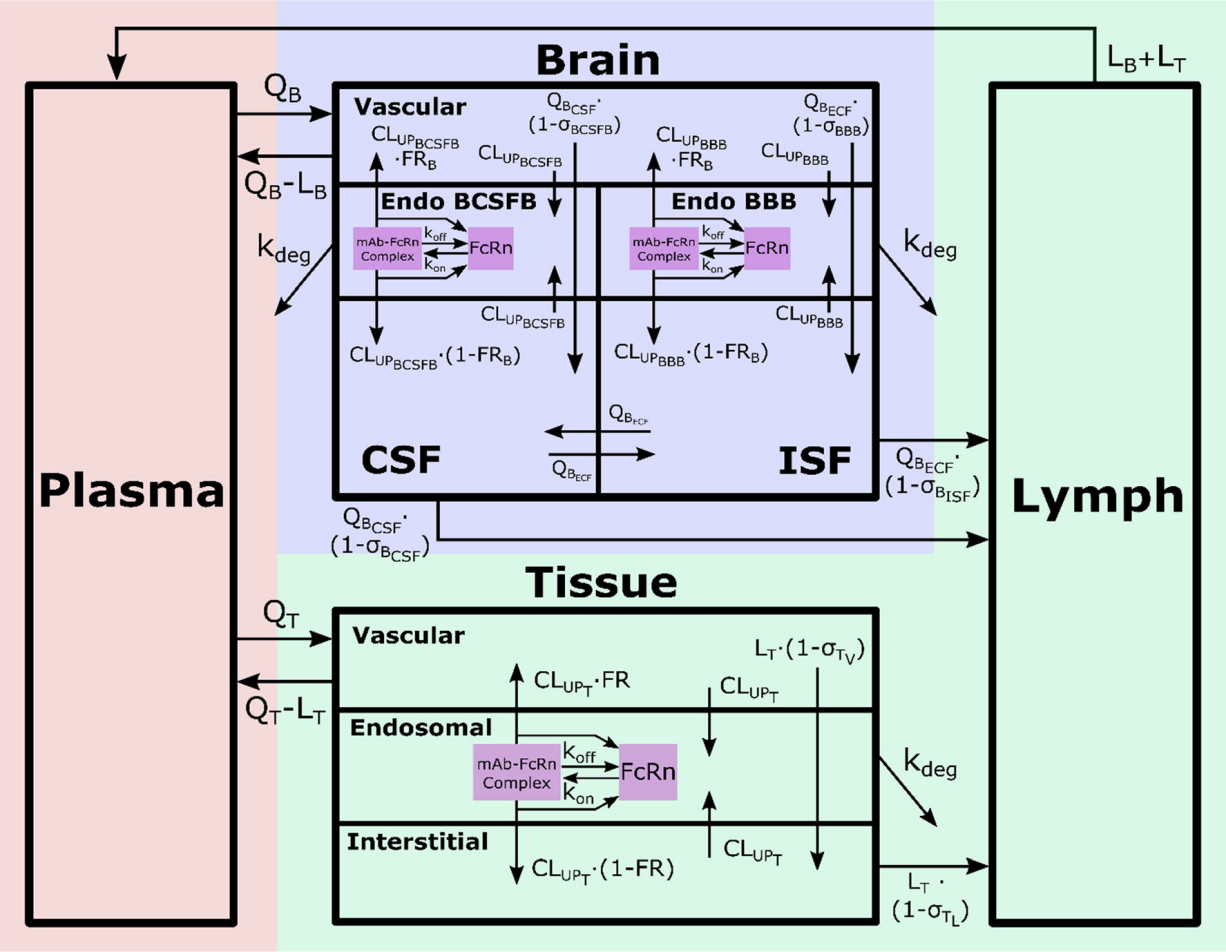
Brain mPBPK model structure. The model contains 16 compartments and three regions: plasma (red), brain (blue) and non-brain tissues (green). Antibodies in plasma flow between brain and non-brain tissues. In tissue vascular, antibody travels transcellularly through the endosomal space or paracellularly by directly entering tissue interstitium. In the endosome, antibody binds FcRn to form an antibody-FcRn complex, which either is taken up into the tissue interstitial space or recycled back to the tissue vasculature. Unbound antibody in the endosome is cleared through lysosomal degradation. Antibody in tissue interstitial space leaves via lymphatic flow as well as via endosomal uptake. Antibody in brain vasculature crosses two brain barriers, BBB and BCSFB. Antibodies that cross the BBB and BCSFB enter the brain ISF and CSF, respectively. Paracellular transport across the BBB and BCSFB is governed by brain vascular reflection coefficients that represent the leakiness of the vasculature space. Transcellular transport across the brain via pinocytosis is described by uptake clearance processes. Antibody in brain endosomal spaces is either recycled via FcRn or eliminated via lysosomal degradation. Antibody in brain ISF and CSF can be cleared back to systemic circulation via the glymphatic system. Diagram was drawn using *Inkscape* (Color figure online)

**Fig. 2:**
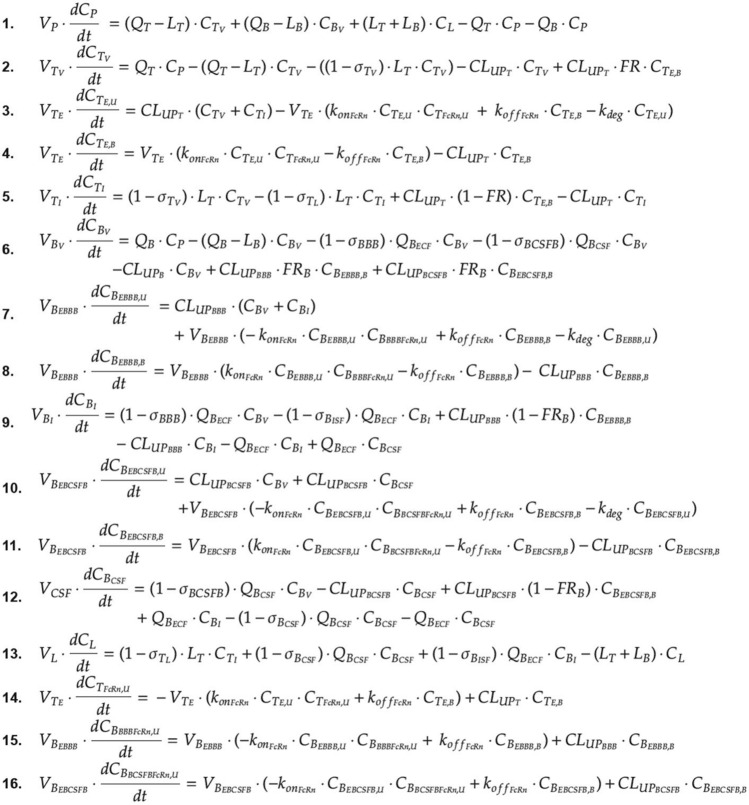
Brain mPBPK model equations. Antibody concentration in (1) plasma, (2) tissue vascular, (3) tissue endosome (unbound), (4) tissue endosome (FcRn-bound), (6) tissue interstitium, (7) brain vascular, (8) BBB endosome (unbound), (9) BBB endosome (FcRn-bound), (11) brain interstitium, (12) BCSFB endosome (unbound), (13) BCSFB endosome (FcRn-bound), (15) brain CSF, (16) lymph. FcRn concentration in (5) tissue endosome, (10) BBB endosome, and (14) BCSFB endosome. Equations written using *Mathcha*

The brain mPBPK model contains 16 differential equations as compared to 100 in the original model. Brain mPBPK model diagram and equations are shown in Figs. 1 and 2, respectively. Antibody concentrations in plasma go to the brain and non-brain tissues at flow rates of Q_B_ and Q_T_, while returning flow rates are lower by a difference of lymph flow (Q_B_–L_B_ and Q_T_–L_T_). In the tissue vascular space, antibody can traverse blood vessel endothelium through paracellular and transcellular routes. Mathematically, paracellular transport is described as a function of the tissue lymph flow and tissue vascular reflection coefficient, $${\mathrm{L}}_{T}\cdot \left(1-{\sigma }_{{T}_{V}}\right)$$. Transcellular transport via pinocytosis is described as the tissue uptake clearance ($${CL}_{{UP}_{T}})$$ from the tissue vasculature into the endosomal space. In the endosomal space, antibody is able to bind to FcRn to form an antibody-FcRn complex and is either taken up into the tissue interstitial space or recycled back to the tissue vasculature, where FR is the fraction of FcRn-bound antibody recycled to the tissue vascular space. Unbound antibody in the endosome is subject to lysosomal degradation at a rate of k_deg_. Antibody then leaves the tissue interstitial space by the lymphatic system, which is a function of tissue lymph flow and tissue lymph reflection coefficient: $${\mathrm{L}}_{T}\cdot \left(1-{\sigma }_{{T}_{L}}\right)$$ or by uptake clearance ($${CL}_{{UP}_{T}})$$ back into endosomal compartment.

Antibody in the brain vasculature space can cross the two brain barriers, BBB and BCSFB. Antibodies transported across the BBB enter the brain ISF, whereas antibodies that move across the BCSFB enter the CSF. The paracellular transport across the BBB and BCSFB is governed by brain vascular reflection coefficients ${\sigma }_{BBB}$ and ${\sigma }_{BCSFB}$. Mathematically, antibody flow across the BBB and BCSFB via paracellular transport is defined by the functions: ${\mathrm{Q}}_{{B}_{ECF}}\cdot \left(1-{\sigma }_{BBB}\right)$ and ${\mathrm{Q}}_{{B}_{CSF}}\cdot \left(1-{\sigma }_{BCSFB}\right)$. Where, ${\mathrm{Q}}_{{B}_{ECF}}$ and ${\mathrm{Q}}_{{B}_{CSF}}$ are the brain extracellular fluid and cerebral spinal fluid (CSF) flow rates. Due to the tight nature of the neurovascular unit, ${\sigma }_{BBB}$ was fixed to one, as originally described, to represent the absence of paracellular transport across the BBB under normal conditions. The paracellular transport of antibodies across the BCSFB is thought to be possible, although quite limited, which is supported by an estimated value of 0.9974 for ${\sigma }_{BCSFB}$ [17]. Transcellular transport across the brain via pinocytosis is described by two uptake clearances, ${CL}_{{UP}_{BBB}}$ and ${CL}_{{UP}_{BCSFB}}$, and the fraction of antibody recycled to the brain vascular space (FR_B_). Antibodies in the brain endosomal spaces that are not bound to FcRn are degraded at a rate of k_deg_. As described by the model, antibodies in the brain ISF and CSF undergo three kinetic processes: (1) uptake into brain endosomal space, (2) flow between brain ISF and CSF compartments, and (3) glymphatic clearance. The flow rate between the ISF and CSF compartments was set to the brain ECF flow rate ${\mathrm{Q}}_{{B}_{ECF}}$. Glymphatic clearance from brain ISF and CSF compartments are described by the following functions:${\mathrm{Q}}_{{B}_{ECF}}\cdot \left(1-{\sigma }_{{B}_{ISF}}\right)$ and ${\mathrm{Q}}_{{B}_{CSF}}\cdot \left(1-{\sigma }_{{B}_{CSF}}\right)$. Lastly, flow from the lymph compartment circulates back into systemic circulation $({L}_{B}+{L}_{T})$.

### Minimal PBPK model performance

**Fig. 3:**
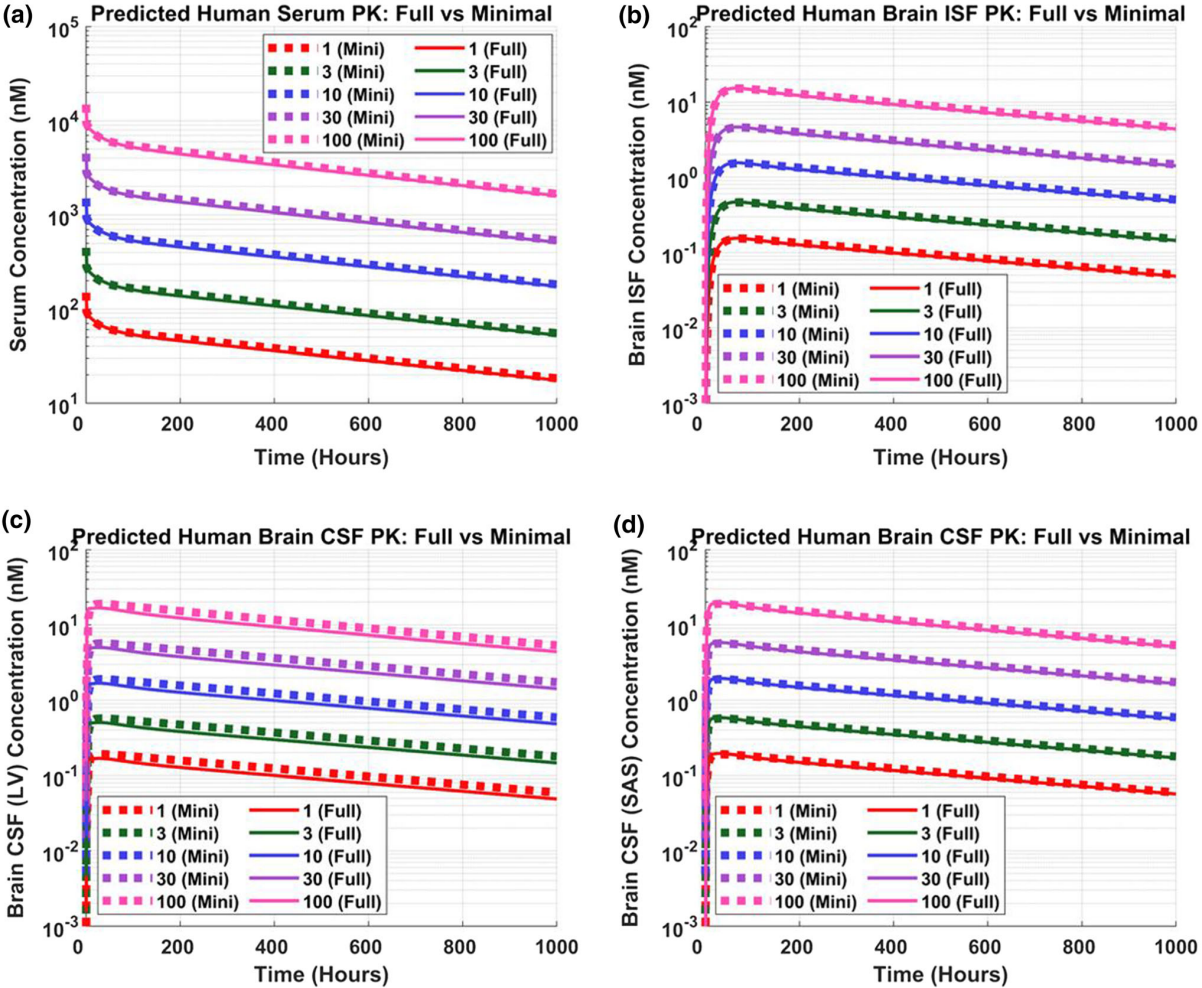
Minimal versus full PBPK model predictions. Antibody concentrations in human **a** serum, **b** brain interstitial fluid (ISF), **c** brain CSF in lateral ventricle (LV), and **d** brain CSF in subarachnoid space (SAS). Five IV doses were simulated 1 (red), 3 (green), 10 (blue), 30 (purple), and 100 (pink) mg/kg for a duration of 1000 h. Dotted and solid lines represent minimal and full PBPK model simulations, respectively (Color figure online)

Model performance was assessed by comparing mPBPK model predictions against simulated data from the full PBPK model. A single ascending dose simulation was performed (1, 3, 10, 30, 100 mg/kg) for human (Fig. 3). Minimal (dotted lines) and full (solid lines) PBPK models predictions nicely overlay across all doses for plasma, ISF, and CSF compartments. CSF PK predictions from the mPBPK model were compared against the full PBPK model for two different CSF compartments, lateral ventricle (Fig. 3c) and subarachnoid space (Fig. 3d). Minimal and full PBPK model predictions also overlay precisely across different preclinical species, mouse (Fig. S1), rat (Fig. S2), and non-human primates (NHP) (Fig. S3).

AUCs were determined for minimal and full PBPK model simulations across all doses and species for plasma, ISF, and CSF compartments. AUC ratios, minimal divided by full, were calculated and are reported in Table 2. AUC ratios range between 0.94 and 1.22, but generally are close to 1, which indicates that PK predictions are comparable between models and the reduction methodology did not significantly impact model predictability. The reduced model seemed to better retain model predictability for rats relative to other species as evidenced by the AUC ratios for three of four compartments being equal to 1. The minimal model slightly overpredicts exposure in the ventricular CSF compartment (AUC ratio = 1.2).

**Table 2:** AUC ratios of minimal vs full PBPK model simulations

	Mouse	Rat	NHP	Human
Plasma	0.96	1.00	1.06	1.05
ISF	0.94	1.00	1.06	1.04
CSF (LV)	1.17	1.19	1.22	1.21
CSF (SAS)	0.96	1.00	1.06	1.04

**Fig. 4:**
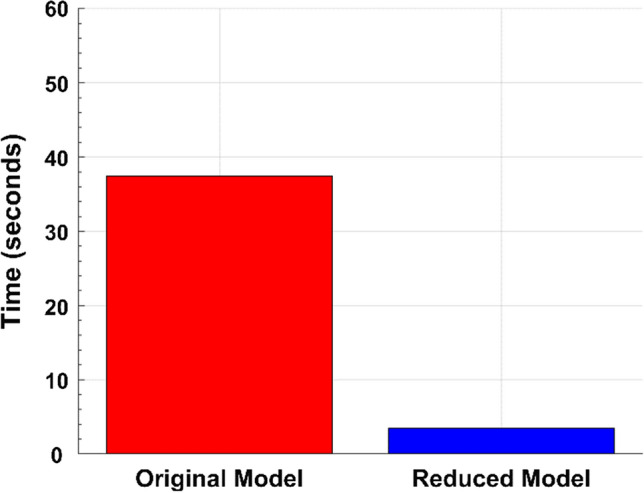
Computational speed improvement. Simulation time differences between the original/full (red) and reduced/minimal (blue) PBPK models. Simulations were performed for 5 doses over 1000 h for each species, using a solver step size of 0.01 h. The differential equation solver used was ode15s using a relative and absolute tolerance of 2.3E−14 and 1E−22, respectively. Each bar represents the average simulation time across four simulations, one simulation per species. Processor: Intel i7-8700K CPU @ 3.70 GHz (Color figure online)

The model reduction method enabled a significant improvement in computational speed. A single simulation on average took 37.5 s for the full model, whereas the minimal model took 3.4 s (Fig. 4). Hence, simulations of the reduced model were approximately 11 times faster. Although this seems like a modest improvement for a single simulation, the computational burden for parameter estimation, Monte Carlo simulations, and other sampling methodologies could significantly improve from the order of days to hours.

### Sensitivity analysis

**Fig. 5:**
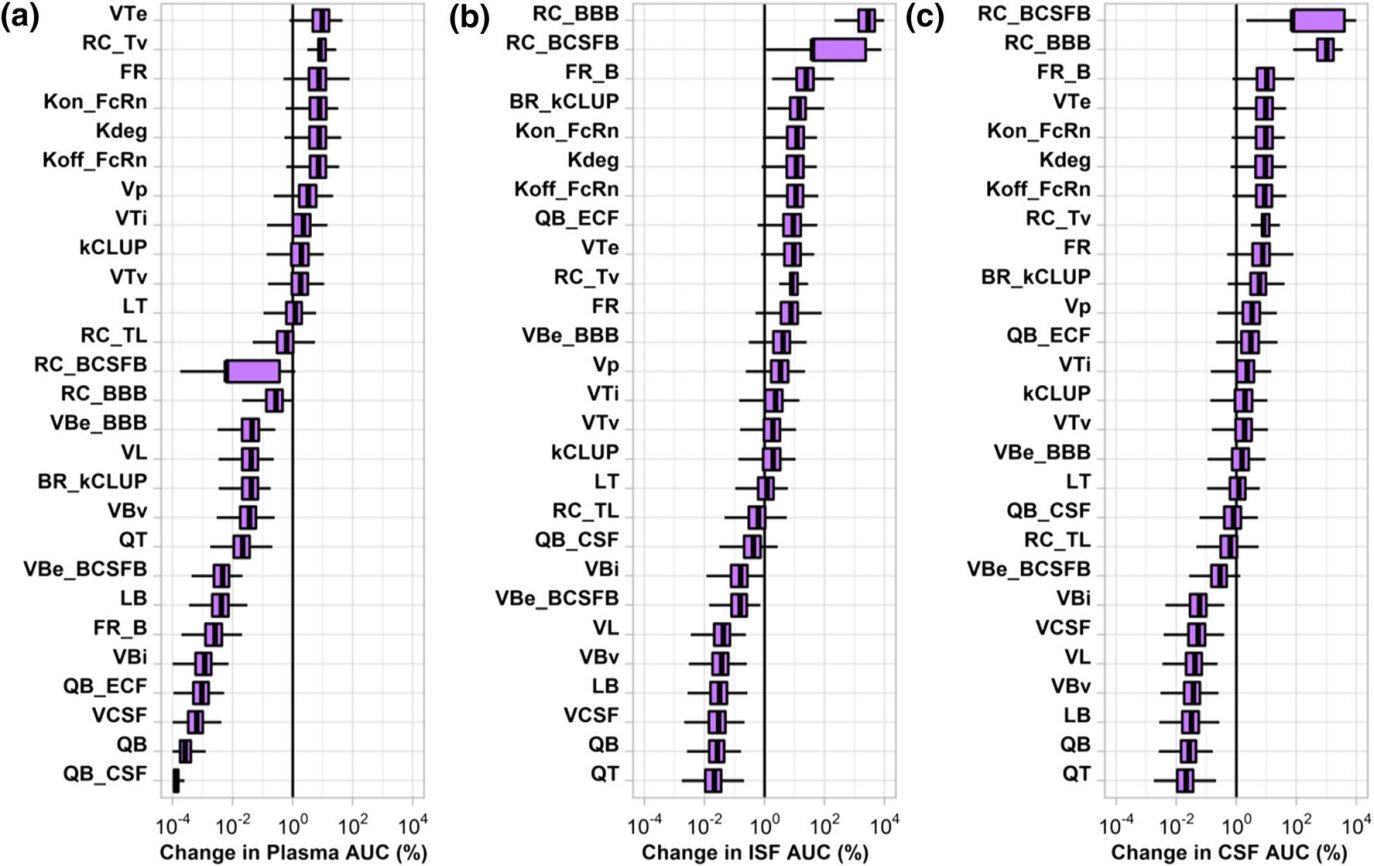
Sensitivity analysis of minimal brain PBPK model for antibody exposure in **a** plasma, **b** brain ISF, and **c** brain CSF. The change in AUC for each parameter is displayed as a box and whisker that represents 1000 simulations. Each parameter (one at a time) was multiplied by a value sampled from a log-normal distribution (µ = 0, σ = 0.25)

The sensitivity of minimal PBPK model parameters for altering plasma (Fig. 5a), brain ISF (Fig. 5b), and brain CSF (Fig. 5c) exposures are displayed in Fig. 5. For antibody exposure in plasma, the most sensitive parameter was the tissue endosomal volume (V_TE_) followed by the reflection coefficient for the tissue vasculature (σ_Tv_). The next sensitive parameters relate to FcRn binding (kon_FcRn_/koff_FcRn_), the fraction of the antibody FcRn complex that recycles (FR), and the endosomal degradation rate (k_deg_). Antibody exposure in brain is governed primarily by two parameters, the BBB and BCSFB reflection coefficients. The BBB reflection coefficient (σ_BBB_) is most sensitive towards brain ISF exposures, whereas the BCSFB reflection coefficient (σ_BCSFB_) is most sensitive towards brain CSF exposures. Other notable sensitive parameters include processes related to FcRn mediated binding and recycling, endosomal volume and degradation rate, and the rate of pinocytosis at brain barriers. Several parameters were insensitive and result in less than a 1% change in AUC (left of the line in Fig. 5). The sensitivity of model parameters in the full PBPK model for altering plasma, brain ISF, and brain CSF exposures are displayed in Supplementary Fig. S4. The rank order and magnitude for the sensitivity of parameters between the minimal and full PBPK models were similar. However, parameters related to the tissue compartment (V_Te_, σ_T_, V_Ti_, V_Tv_) were more sensitive in the minimal model compared to the full model. This could be due to the fact that the tissue compartment in the minimal model represents all combined tissues and the tissue compartment used in the sensitivity analysis of the full model only represents a single tissue.

## Discussion

We have constructed a mPBPK model with detailed brain physiology. We have shown that the model reduction methodology did not lead to any appreciable change in model predictability, since the predicted plasma and brain concentration–time profiles from the minimal model are comparable to the full model. The minimal model also displayed significant improvements in computational speed, which could save time when conducting parameter estimation, sensitivity analyses, Monte Carlo simulations, population analyses, and other methodologies. The simplicity of the mPBPK model could improve upon model transparency, ease of understanding, and future utilization. A minimalistic pharmacokinetic model could enable an easier integration with platform quantitative systems pharmacology (QSP) models of neurological disease, which have been increasing in popularity over the last few years [19]. The model is available as MATLAB code and a SimBiology project file (MATLAB version 2020b). For SimBiology, the parameter sets for the individual species are captured as separate variants while dosing is handled through the *Dose* tab. Drug specific parameters can be added as a new variant and combined with the species variant of interest for simulations.

Model applications include a priori predictions of antibody pharmacokinetics in plasma and brain in mouse, rat, monkey, and human, which could be used to evaluate drug exposure differences for various dosing regimens. Moreover, the model could be expanded to describe in detail distribution into any other tissue/organ of choice which was included in the full model. Engineering Fc regions to extend the half-life of circulating antibodies has become a popular method for increasing drug exposure and decreasing the frequency of drug administration [20]. The role of differences in FcRn binding due these mutations on antibody half-life could be evaluated using the model. However, FcRn binding improvements do not always translate into longer half-life as other physicochemical characteristics that govern endosomal trafficking dynamics also play a role in drug elimination [21, 22]. This is a current limitation of the model as improving FcRn affinity will always result in PK predictions with improved half-life. Understanding the structural and physicochemical characteristics of antibodies that govern uptake into the endosome, FcRn binding and recycling, and endosomal degradation would provide insights into inter-antibody pharmacokinetic differences. Another consideration is the competition for binding to FcRn by endogenous IgG. There is a lack of experimental measurements for the concentration of FcRn, which was originally estimated, and the fraction of antibody recycled via FcRn [18]. Additionally, there is some uncertainty around the parameter for endosomal volume, which could range from 0.034 to 0.5% of total tissue volume [23, 24]. These uncertainties should be considered when including endogenous IgG competition.

Currently, the model is only applicable to describe the pharmacokinetics of antibodies. The model could be repurposed for other biologics, however there are a few points to consider. First, antibodies are able to bind to FcRn to prevent degradation via the lysosome, which may not be applicable to other biologics. Therefore, one would need to determine potential interactions with FcRn or other recycling mechanisms and potential differences in the endosomal degradation rate. Second, biologics that are small in molecular weight could be subject to clearance via glomerular filtration, which has been described in a recent paper investigating clearance differences between antibody fragments and IgG in mice [25]. Biologics smaller than albumin (66.5 kDa) begin to exhibit enhanced clearance via glomerular filtration [26]. Third, the vascular permeability of biologics could be different than antibodies. Therefore, parameters that govern the uptake of antibody into tissues, such as the tissue and brain reflection coefficients, would have to be re-estimated. In future, as data accumulates, these parameters could be defined as a function of molecular size and other relevant physicochemical characteristics.

One of the most significant applications is expanding the mPBPK brain model to include targets of interest, which would expand model utility and enable predictions to understand target engagement, determine efficacious dosing regimens for clinical studies, support affinity optimization by understanding desirable affinity ranges, and evaluate levels of target engagement amongst several drug candidates. Expanding the model to include target-mediated processes introduces additional parameters that need to be defined, such as target expression and turnover as well as antibody-target association and dissociation rates [27]. Various experimental technologies, such as Biacore and KinExA, can be used to measure antibody affinity [28]. Careful consideration should be taken in the experimental designs and use of these affinity measurements as there can be a significant amount of variability between/within assays and potential disconnects between in vitro measurements and in vivo observations. Common drug targets for neurodegenerative diseases are proteins that self-aggregate. Applications could include evaluating relative target engagement to monomeric, oligomeric, and insoluble species. The target of interest could exist in plasma, non-brain tissues, brain ISF and CSF. If the target exists in plasma, the model should also be expanded to include the target in plasma as well as brain and tissue vascular compartments. The target and antibody target complex could follow the same kinetic processes as antibodies or a simplifying assumption could be implemented where the target and antibody target complex don’t distribute between compartments. The antibody target complex could follow the same elimination as a typical antibody, which is often the case for soluble targets that have a relatively lower molecular weight than an antibody. For membrane targets, a key parameter to determine is the antibody-receptor complex internalization rate as the target could impact antibody elimination.

Multiple clinical trials investigating therapeutic antibodies for the treatment of neurodegenerative disease have used concentrations of antibody and target engagement in the CSF as a surrogate for expected concentrations and engagement in the brain [10]. However, this may not be an entirely appropriate assumption as there could be differences in drug pharmacokinetics and target dynamics (expression and turnover) in brain ISF, the site of drug action, compared to CSF. Since it is experimentally impractical to sample drug concentrations and engagement in the human brain, a PBPK model of the brain expanded to include the target of interest enables drug exposure and target engagement predictions in an otherwise unobservable compartment. The minimal PBPK model presented here could be expanded to include drug targets to support preclinical and clinical drug development programs investigating antibody therapeutics for the treatment of neurological diseases.

## Supplementary Information

Below is the link to the electronic supplementary material.

Supplementary file1 (ZIP 44510 kb)

Supplementary file2 (ZIP 144509 kb)

Supplementary file3 (DOCX 2763 kb)
