# Supplementary material for: Minimal brain PBPK model to support the preclinical and clinical development of antibody therapeutics for CNS diseases
Source: J Pharmacokinet Pharmacodyn. 2021 Aug 10;48(6):861–71. doi: 10.1007/s10928-021-09776-7 (PMC8604880; doi:10.1007/s10928-021-09776-7)
Supplement: Supplementary file 1 — Supplementary file1 (ZIP 44510 kb) [file 10928_2021_9776_MOESM1_ESM.zip › Brain mPBPK Model/Brain mPBPK Antibody Equations.pdf]

1. $$V_P \cdot \frac{dC_P}{dt} = (Q_T - L_T) \cdot C_{T_V} + (Q_B - L_B) \cdot C_{B_V} + (L_T + L_B) \cdot C_L - Q_T \cdot C_P - Q_B \cdot C_P$$

2. $$V_{T_V} \cdot \frac{dC_{T_V}}{dt} = Q_T \cdot C_P - (Q_T - L_T) \cdot C_{T_V} - ((1 - \sigma_{T_V}) \cdot L_T \cdot C_{T_V}) - CL_{UP_T} \cdot C_{T_V} + CL_{UP_T} \cdot FR \cdot C_{T_{E,B}}$$

3. $$V_{T_E} \cdot \frac{dC_{T_{E,U}}}{dt} = CL_{UP_T} \cdot (C_{T_V} + C_{T_I}) - V_{T_E} \cdot (k_{on_{FcRn}} \cdot C_{T_{E,U}} \cdot C_{T_{FcRn,U}} + k_{off_{FcRn}} \cdot C_{T_{E,B}} - k_{deg} \cdot C_{T_{E,U}})$$

4. $$V_{T_E} \cdot \frac{dC_{T_{E,B}}}{dt} = V_{T_E} \cdot (k_{on_{FcRn}} \cdot C_{T_{E,U}} \cdot C_{T_{FcRn,U}} - k_{off_{FcRn}} \cdot C_{T_{E,B}}) - CL_{UP_T} \cdot C_{T_{E,B}}$$

5. $$V_{T_I} \cdot \frac{dC_{T_I}}{dt} = (1 - \sigma_{T_V}) \cdot L_T \cdot C_{T_V} - (1 - \sigma_{T_L}) \cdot L_T \cdot C_{T_I} + CL_{UP_T} \cdot (1 - FR) \cdot C_{T_{E,B}} - CL_{UP_T} \cdot C_{T_I}$$

6. $$V_{B_V} \cdot \frac{dC_{B_V}}{dt} = Q_B \cdot C_P - (Q_B - L_B) \cdot C_{B_V} - (1 - \sigma_{BBB}) \cdot Q_{B_{ECF}} \cdot C_{B_V} - (1 - \sigma_{BCSFB}) \cdot Q_{B_{CSF}} \cdot C_{B_V}$$
$$-CL_{UP_B} \cdot C_{B_V} + CL_{UP_{BBB}} \cdot FR_B \cdot C_{B_{EBBB,B}} + CL_{UP_{BCSFB}} \cdot FR_B \cdot C_{B_{EBCSFB,B}}$$

7. $$V_{B_{EBBB}} \cdot \frac{dC_{B_{EBBB,U}}}{dt} = CL_{UP_{BBB}} \cdot (C_{B_V} + C_{B_I})$$
$$+ V_{B_{EBBB}} \cdot (- k_{on_{FcRn}} \cdot C_{B_{EBBB,U}} \cdot C_{B_{BBBFcRn,U}} + k_{off_{FcRn}} \cdot C_{B_{EBBB,B}} - k_{deg} \cdot C_{B_{EBBB,U}})$$

8. $$V_{B_{EBBB}} \cdot \frac{dC_{B_{EBBB,B}}}{dt} = V_{B_{EBBB}} \cdot (k_{on_{FcRn}} \cdot C_{B_{EBBB,U}} \cdot C_{B_{BBBFcRn,U}} - k_{off_{FcRn}} \cdot C_{B_{EBBB,B}}) - CL_{UP_{BBB}} \cdot C_{B_{EBBB,B}}$$

9. $$V_{B_I} \cdot \frac{dC_{B_I}}{dt} = (1 - \sigma_{BBB}) \cdot Q_{B_{ECF}} \cdot C_{B_V} - (1 - \sigma_{B_{ISF}}) \cdot Q_{B_{ECF}} \cdot C_{B_I} + CL_{UP_{BBB}} \cdot (1 - FR_B) \cdot C_{B_{EBBB,B}}$$
$$- CL_{UP_{BBB}} \cdot C_{B_I} - Q_{B_{ECF}} \cdot C_{B_I} + Q_{B_{ECF}} \cdot C_{B_{CSF}}$$

10. $$V_{B_{EBCSFB}} \cdot \frac{dC_{B_{EBCSFB,U}}}{dt} = CL_{UP_{BCSFB}} \cdot C_{B_V} + CL_{UP_{BCSFB}} \cdot C_{B_{CSF}}$$
$$+V_{B_{EBCSFB}} \cdot (-k_{on_{FcRn}} \cdot C_{B_{EBCSFB,U}} \cdot C_{B_{BCSFBFcRn,U}} + k_{off_{FcRn}} \cdot C_{B_{EBCSFB,B}} - k_{deg} \cdot C_{B_{EBCSFB,U}})$$

11. $$V_{B_{EBCSFB}} \cdot \frac{dC_{B_{EBCSFB,B}}}{dt} = V_{B_{EBCSFB}} \cdot (k_{on_{FcRn}} \cdot C_{B_{EBCSFB,U}} \cdot C_{B_{BCSFBFcRn,U}} - k_{off_{FcRn}} \cdot C_{B_{EBCSFB,B}}) - CL_{UP_{BCSFB}} \cdot C_{B_{EBCSFB,B}}$$

12. $$V_{CSF} \cdot \frac{dC_{B_{CSF}}}{dt} = (1 - \sigma_{BCSFB}) \cdot Q_{B_{CSF}} \cdot C_{B_V} - CL_{UP_{BCSFB}} \cdot C_{B_{CSF}} + CL_{UP_{BCSFB}} \cdot (1 - FR_B) \cdot C_{B_{EBCSFB,B}}$$
$$+ Q_{B_{ECF}} \cdot C_{B_I} - (1 - \sigma_{B_{CSF}}) \cdot Q_{B_{CSF}} \cdot C_{B_{CSF}} - Q_{B_{ECF}} \cdot C_{B_{CSF}}$$

13. $$V_L \cdot \frac{dC_L}{dt} = (1 - \sigma_{T_L}) \cdot L_T \cdot C_{T_I} + (1 - \sigma_{B_{CSF}}) \cdot Q_{B_{CSF}} \cdot C_{B_{CSF}} + (1 - \sigma_{B_{ISF}}) \cdot Q_{B_{ECF}} \cdot C_{B_I} - (L_T + L_B) \cdot C_L$$

14. $$V_{T_E} \cdot \frac{dC_{T_{FcRn,U}}}{dt} = - V_{T_E} \cdot (k_{on_{FcRn}} \cdot C_{T_{E,U}} \cdot C_{T_{FcRn,U}} + k_{off_{FcRn}} \cdot C_{T_{E,B}}) + CL_{UP_T} \cdot C_{T_{E,B}}$$

15. $$V_{B_{EBBB}} \cdot \frac{dC_{B_{BBBFcRn,U}}}{dt} = V_{B_{EBBB}} \cdot (-k_{on_{FcRn}} \cdot C_{B_{EBBB,U}} \cdot C_{B_{BBBFcRn,U}} + k_{off_{FcRn}} \cdot C_{B_{EBBB,B}}) + CL_{UP_{BBB}} \cdot C_{B_{EBBB,B}}$$

16. $$V_{B_{EBCSFB}} \cdot \frac{dC_{B_{BCSFBFcRn,U}}}{dt} = V_{B_{EBCSFB}} \cdot (-k_{on_{FcRn}} \cdot C_{B_{EBCSFB,U}} \cdot C_{B_{BCSFBFcRn,U}} + k_{off_{FcRn}} \cdot C_{B_{EBCSFB,B}}) + CL_{UP_{BCSFB}} \cdot C_{B_{EBCSFB,B}}$$
