# Supplementary material for: Minimal brain PBPK model to support the preclinical and clinical development of antibody therapeutics for CNS diseases
Source: J Pharmacokinet Pharmacodyn. 2021 Aug 10;48(6):861–71. doi: 10.1007/s10928-021-09776-7 (PMC8604880; doi:10.1007/s10928-021-09776-7)
Supplement: Supplementary file 3 — Supplementary file3 (DOCX 2763 kb) [file 10928_2021_9776_MOESM3_ESM.docx]

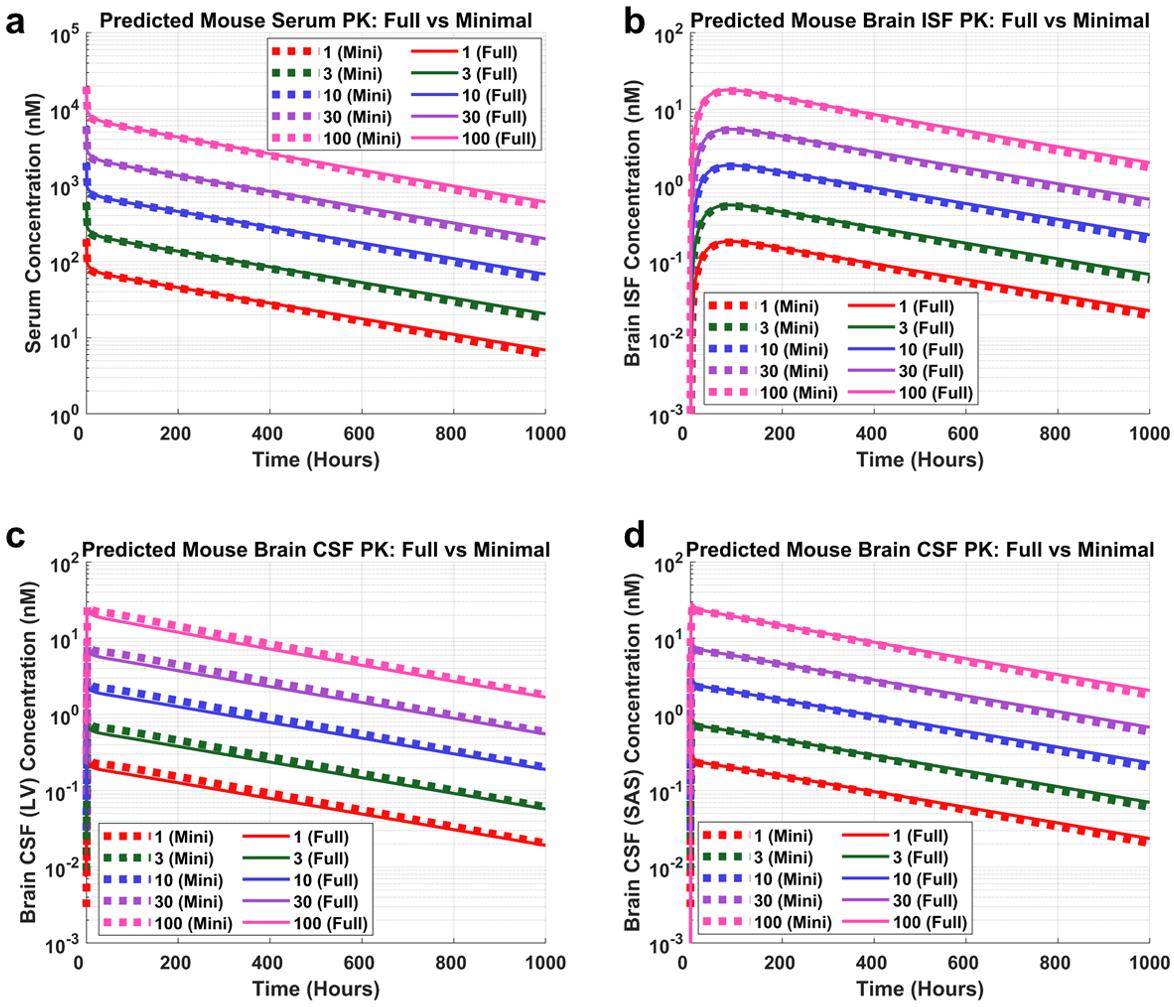


**Fig. S1** Minimal versus original (full) PBPK model predictions for antibody concentrations in mouse (a) serum, (b) brain interstitial fluid (ISF), (c) brain CSF in lateral ventricle (LV), and (d) brain CSF in subarachnoid space (SAS). Five IV doses were simulated 1 (red), 3 (green), 10 (blue), 30 (purple), and 100 (pink) mg/kg for a duration of 1000 hours. Dotted and solid lines represent minimal and full PBPK model simulations, respectively.


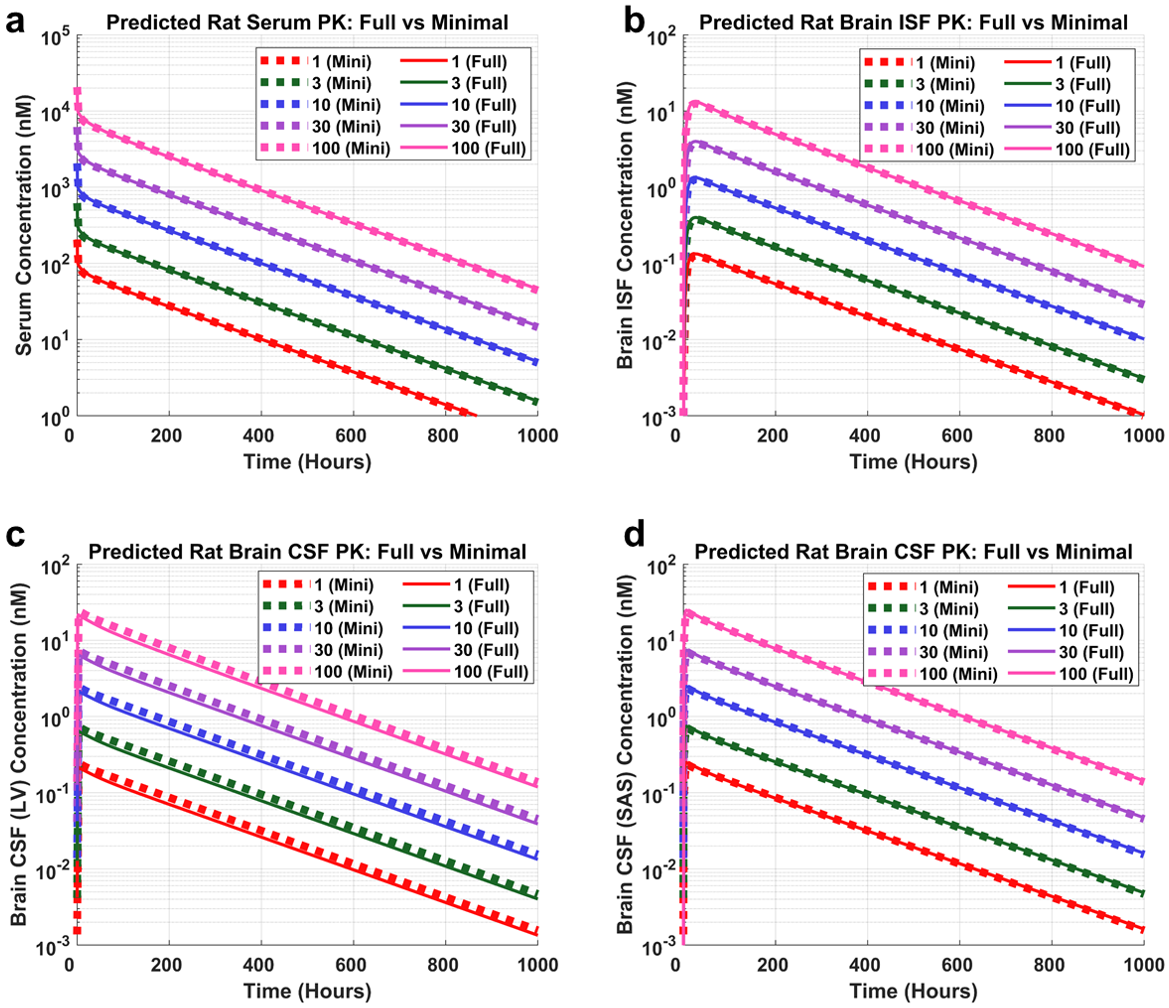


**Fig. S2** Minimal versus original (full) PBPK model predictions for antibody concentrations in rat (a) serum, (b) brain interstitial fluid (ISF), (c) brain CSF in lateral ventricle (LV), and (d) brain CSF in subarachnoid space (SAS). Five IV doses were simulated 1 (red), 3 (green), 10 (blue), 30 (purple), and 100 (pink) mg/kg for a duration of 1000 hours. Dotted and solid lines represent minimal and full PBPK model simulations, respectively.


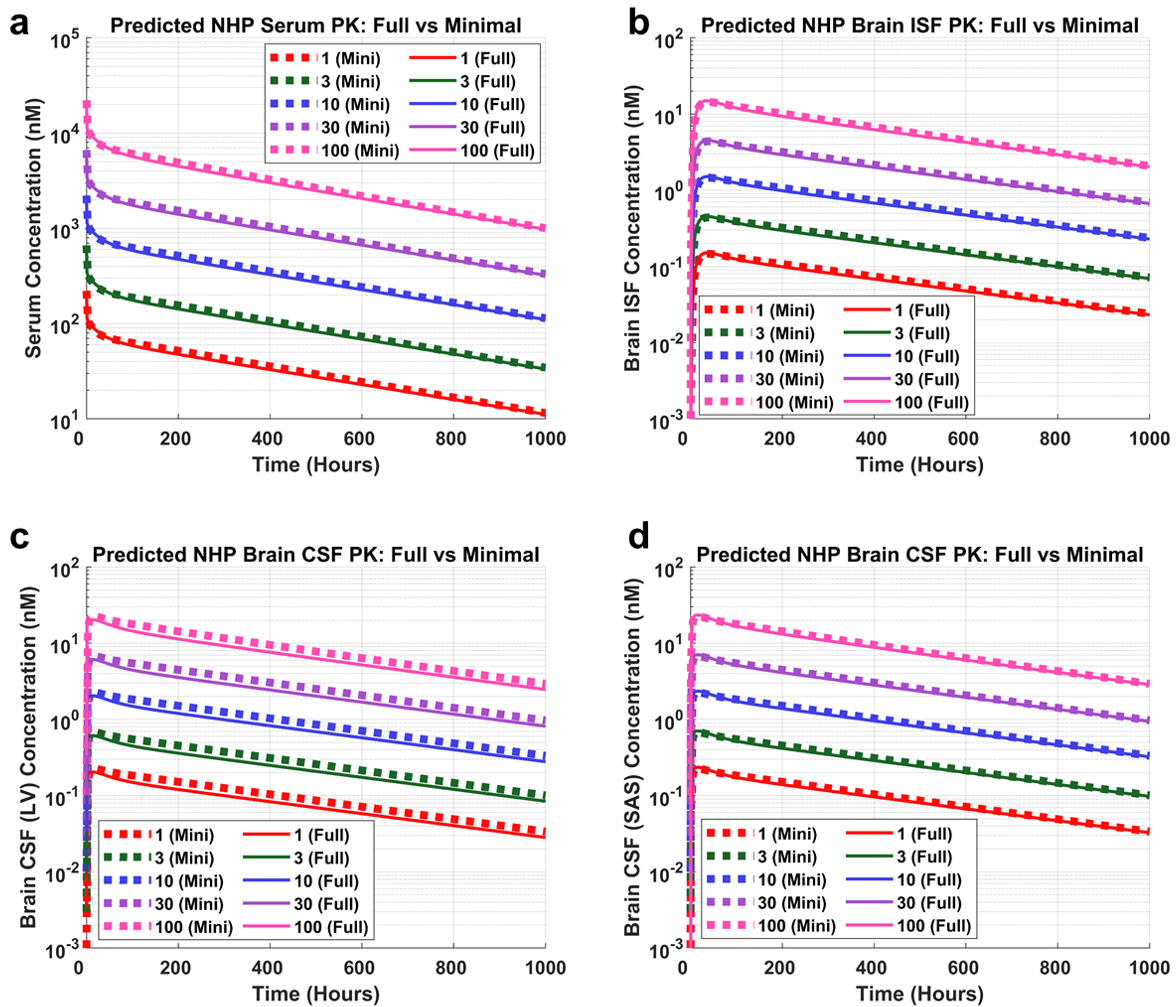


**Fig. S3** Minimal versus original (full) PBPK model predictions for antibody concentrations in non-human primates (a) serum, (b) brain interstitial fluid (ISF), (c) brain CSF in lateral ventricle (LV), and (d) brain CSF in subarachnoid space (SAS). Five IV doses were simulated 1 (red), 3 (green), 10 (blue), 30 (purple), and 100 (pink) mg/kg for a duration of 1000 hours. Dotted and solid lines represent minimal and full PBPK model simulations, respectively.


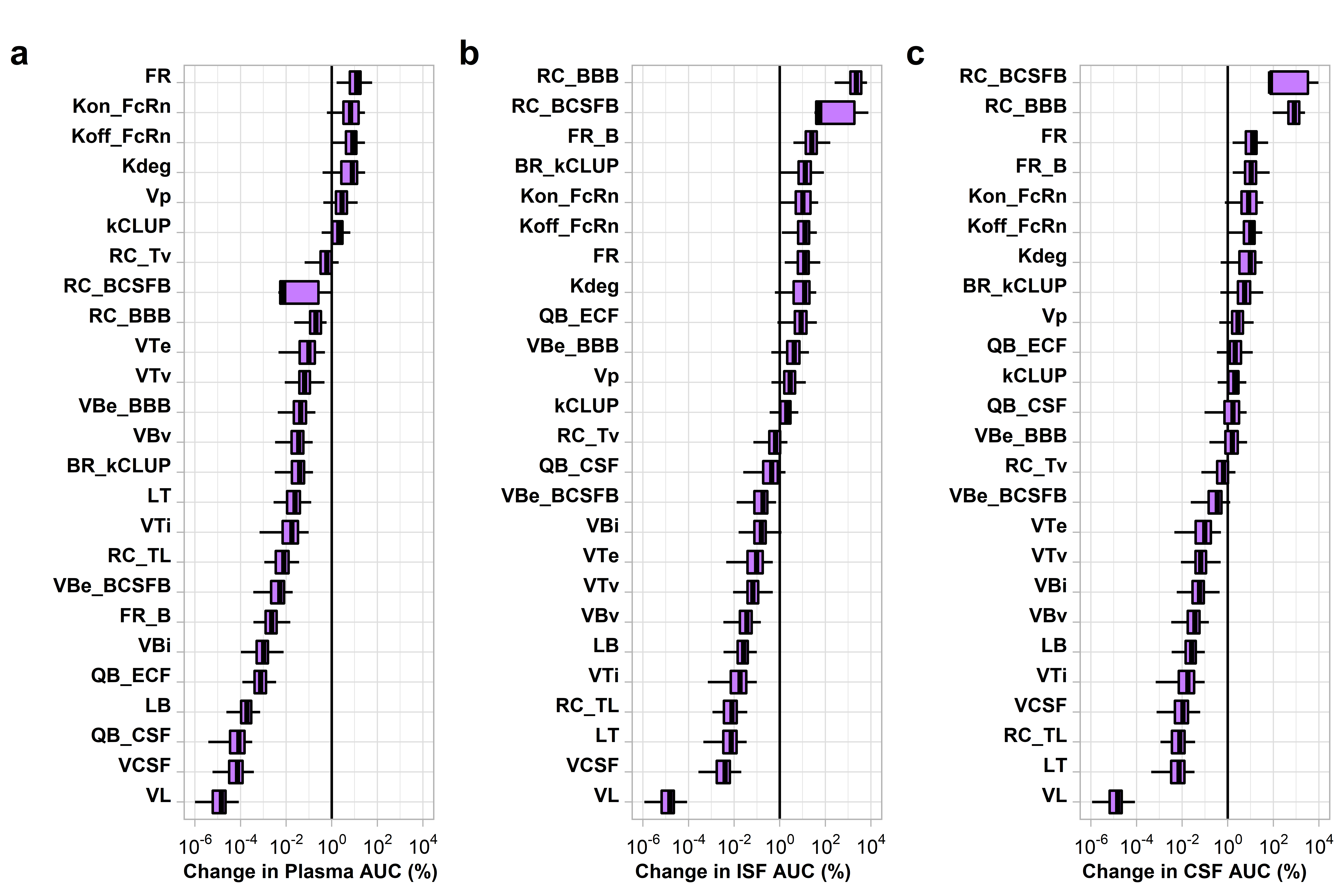


**Fig. S4** Sensitivity analysis of full brain PBPK model for antibody exposure in (a) plasma, (b) brain ISF, and (c) brain CSF. The change in AUC for each parameter is displayed as a box and whisker that represents 1000 simulations. Each parameter (one at a time) was multiplied by a value sampled from a log-normal distribution (µ = 0, σ = 0.25).
